# Integrating Basic Sciences into Dental Education: A Comparative Narrative Review of Curricular Approaches Across Different Countries

**DOI:** 10.3390/dj14080529

**Published:** 2026-08-19

**Authors:** Ghazal Ek, Vår Kristidatter Syversen, Qalbi Khan, Tor Paaske Utheim, Amer Sehic

**Affiliations:** 1 Institute of Oral Biology, Faculty of Dentistry, University of Oslo, 0316 Oslo, Norway; ghazale@student.odont.uio.no (G.E.);; 2 Department of Medical Biochemistry, Oslo University Hospital, 0424 Oslo, Norway; 3 Department of Ophthalmology, Sørlandet Hospital Trust, 4838 Arendal, Norway; 4 Department of Plastic and Reconstructive Surgery, Oslo University Hospital, 0424 Oslo, Norway

**Keywords:** basic science education, curriculum integration, dental education, knowledge retention, pedagogy

## Abstract

**Background/Objectives:** Basic sciences form the conceptual foundation of dentistry, yet their delivery in dental curricula varies worldwide. As demographic shifts, growing healthcare complexity and scientific advances continue to reshape the dental profession, renewed attention to how these subjects are taught has become essential. This comparative narrative review aims to examine different approaches to basic science education in dentistry, exploring variation in curricular structure, depth, timing and pedagogical strategies across diverse dental schools. Particular emphasis is placed on models of vertical, horizontal and spiral integration, the alignment of basic sciences with clinical training and the implications of instructional design for long-term knowledge retention and professional competence. **Methods:** PubMed and Google Scholar were searched from November 2025 to June 2026 using terms related to dental education, curriculum integration and geographic regions. Sources were selected based on predefined relevance criteria rather than through systematic screening. Published literature was supplemented by structured outreach to faculty representatives using a standardised set of questions. Of the 25 dental schools contacted, nine responded, and five were included. **Results:** Across the programs examined, a structure of approximately two preclinical years followed by clinical training remained the most common. Early clinical exposure appeared to strengthen student confidence and professional identity. Heavy workload and curricular overload were recurring challenges in the dental curricula examined. **Conclusions:** The findings suggest that although curriculum integration is widely recognised as essential, no single model appears to be universally superior. Its effectiveness likely depends on the quality of implementation and faculty coordination. However, the lack of independent evaluation studies limits the ability to draw conclusions about the long-term effectiveness of different educational approaches. The review highlights the need for independent and systematic evaluation of dental curricula.

## 1. The Evolving Landscape of Dental Education

Educating dental students is an increasingly complex endeavour that requires balancing scientific knowledge, technical competence and patient-centred care. Modern dentistry operates in a rapidly evolving healthcare landscape characterised by demographic shifts, technological advances and an increase in multimorbidity and polypharmacy among patients, largely driven by global population ageing [1,2,3]. Therefore, future dentists must understand the basic sciences sufficiently well to manage medically compromised patients safely, and this has renewed interest in how dental curricula integrate basic science education with clinical training [2]. However, knowledge retention is often poor. Dental and medical students may forget up to one-third of their basic science knowledge in one year, with retention falling below 50% in the second year after completing coursework [4]. This decline has been attributed to limited integration, the gap between learning and clinical application, and students’ perceptions that preclinical subjects lack direct relevance to their practice, which promotes short-term memorisation [2].

Traditionally, dental education in many countries has devoted two years to basic science courses before gradually transitioning to clinical practice [5]. While this ensures substantial exposure to the basic sciences, it is often criticised for creating a disconnect between theory and practice [6], and this separation has been shown to reduce motivation and increase cognitive overload [1,7]. This is consistent with the learning sciences, which show that knowledge acquired without relevant context often remains inert and difficult to transfer to real-world practice [8]. Bertolami (2001) describes learning large amounts of basic science “out of context” as pedagogically ineffective, arguing that a rigid, fragmented structure contributes to stress, demoralisation, and an overall negative student experience [1]. In contrast, early clinical exposure appears to foster professional socialisation, helping students engage with patients more confidently and supporting the development of professional identity [9].

In response, many institutions have implemented integrated curriculum models [10], commonly described as horizontal, vertical and spiral integration [11,12]. Strategies such as early patient contact, case-based learning (CBL), problem-based learning (PBL) and simulation improve students’ ability to apply theory in clinical settings and enhance their clinical reasoning [13,14]. Theoretical explanations for these models and strategies remain debated. Knowledge encapsulation theory suggests that basic science knowledge is gradually transformed into clinically relevant “illness scripts” [15], whereas another model proposes that basic science and clinical knowledge are separate cognitive domains that contribute independently to diagnostic reasoning [16]. Empirical evidence more strongly supports knowledge encapsulation theory [17], indicating that basic science knowledge tends to be integrated into clinical reasoning patterns rather than remain a completely separate domain.

Given the considerable variation in the global structure of dental education, there is a valuable opportunity to learn from different approaches. Dental curricula vary in length, organisation, timing of clinical exposure and the degree of integration between basic sciences and clinical training. In the United States, dental education is a four-year graduate-entry program (DDS/DMD) pursued after a bachelor’s degree [18]. In contrast, most European countries offer a five-year integrated undergraduate model [19]. Regardless of the structural model, there is a shift towards competency-based education (CBE) [20]. This approach emphasises skill development over fixed timeframes, allowing greater flexibility and more individualised progress for the student.

Despite this diversity, few studies have compared how different countries organise the relationship between basic science and clinical training in dentistry. What is lacking is not agreement on the importance of basic sciences, but evidence regarding how much should be taught, when and with what emphasis [21]. Considering this, there is a need for comparative overviews that examine this issue because dental education faces new challenges and higher expectations for interprofessional collaboration. The purpose of this review is therefore to compare different approaches to dental curriculum design, with particular emphasis on the relationship between basic sciences and clinical learning.

## 2. Methods

### 2.1. Study Design

This study compares how different dental programs structure the relationship between basic sciences and clinical training and examines similarities and differences among them. The curricula are described rather than measured, and the depth and format of the available documentation vary considerably across institutions and regions. Therefore, we provide a narrative comparative review that draws on published research as well as institutional documents and personal communication.

### 2.2. Literature Search and Source Selection

Searches were performed in PubMed and Google Scholar between November 2025 and June 2026. No restrictions were applied regarding publication year. Publications in English, Norwegian, Swedish, Danish and German were considered eligible.

Searches combined terms related to dental education and curriculum integration, including “Dental education”, “Curriculum integration”, “Knowledge retention”, “Integration strategies”, “Dental curriculum” and “Basic science”. To ensure representation from different countries and regions, these terms were combined with geographic identifiers for North America, Europe, Asia, the Middle East and Africa. Representative search strings included “Dental education” AND “Basic science” AND “Europe” AND “Curriculum integration” and “Knowledge retention” AND “Dental education”.

Given the narrative and exploratory nature of this review, formal screening based on systematic review methodology was not performed. However, publications were selected using predefined relevance criteria. Sources were considered eligible if they described or evaluated dental curriculum structure, the sequencing of basic science, integration strategies and knowledge retention. In addition, sources addressing the importance of basic science in dental education, as well as studies from medicine and other health professions, were also included when their findings were considered applicable. Sources were excluded if they were published in a language other than those specified above, if the full text was unavailable or if they focused only on postgraduate or specialist training rather than undergraduate education.

Reference lists of key articles were also reviewed, and relevant sources were included. The final reference list comprised 46 peer-reviewed publications, three articles from professional dental magazines, 17 institutional websites or documents and personal communication with representatives from five dental schools. These different types of sources vary in reliability and credibility, and it is important that readers are aware of and consider these differences when interpreting the findings.

### 2.3. Institutional Outreach

When published literature was limited, direct outreach to faculty representatives was used to supplement the available evidence. The selection of institutions was largely pragmatic. Because the authors were most familiar with the Nordic educational systems, all dental schools in the Nordic countries were contacted. Several dental schools in the United States were then approached, although no responses were received. Additional European institutions were contacted through existing academic collaborations established by a faculty member at the authors’ institution. In total, 25 dental schools were contacted. Responses were received from nine institutions, five of which provided sufficient information to be included in the review.

The same standardised set of questions was used for all institutional contacts (Supplementary Material Table S1). Minor adjustments to wording and format were made depending on whether communication occurred via email, Microsoft Teams or a Word document, but the topics and requested information remained consistent across institutions.

Information obtained through this outreach was verified by the relevant institutional representatives before inclusion. Publications recommended by the institutions were included when relevant to the study. Because only factual information about institutional curricula was collected and no personal or sensitive data were obtained, formal ethical approval was not deemed necessary.

While this outreach offered valuable, first-hand perspectives, it also introduces some subjectivity. The information shared reflects the views and priorities of individual contacts and has not been independently verified beyond the authors’ own review.

### 2.4. Methodological Considerations

The institutions included in this review were not selected according to a predefined sampling framework. Rather, they reflect the available literature identified through the search process. Searches were performed using broad regional terms, such as “Dental curriculum Middle East” or “Dental education Africa”, rather than targeting specific institutions in advance. The institutions discussed are therefore those for which sufficient and accessible documentation was available, rather than a representative sample of all dental schools in a given country or region. Institutions that did not respond to outreach, or for which no suitable contact could be identified, are consequently underrepresented. The same applies to other institutions, including potentially innovative programs that may not have been identified during the search.

Accordingly, this review should be understood as a broad comparative overview rather than as a systematic or comprehensive account of dental education across different contexts. Its value lies in identifying patterns, tensions and gaps in a diverse range of settings, while also highlighting areas where more rigorous and up-to-date evaluation is needed. A comparative summary of curricular features across some of the included institutions is provided in Supplementary Table S1.

## 3. Theoretical Approaches to Curriculum Integration

Curriculum integration can be defined as an educational approach in which teaching and learning activities are organised to help students make meaningful connections between knowledge, skills and attitudes across disciplines and over time [22]. An integrated curriculum is designed to give learners opportunities to combine knowledge and skills from different disciplines to address complex problems and promote higher-order learning [12,22]. In dentistry, this approach is especially important because clinical practice requires the simultaneous application of basic science knowledge, clinical reasoning and technical skills [20]. One major challenge in traditional dental curricula is the pronounced division between preclinical and clinical phases, which may limit students’ ability to apply theoretical knowledge in clinical contexts [23].

Curriculum integration has emerged as a key concept in educational reform and is now widely adopted across health professions education, including medicine, dentistry and pharmacy [11,12]. Instead of emphasising discipline-based teaching, integrated curricula aim to align learning objectives, teaching activities and assessments with the realities of professional practice. This shift reflects the recognition that healthcare professionals must integrate knowledge from multiple domains to deliver safe and effective patient care [12,23].

**Figure 1:**
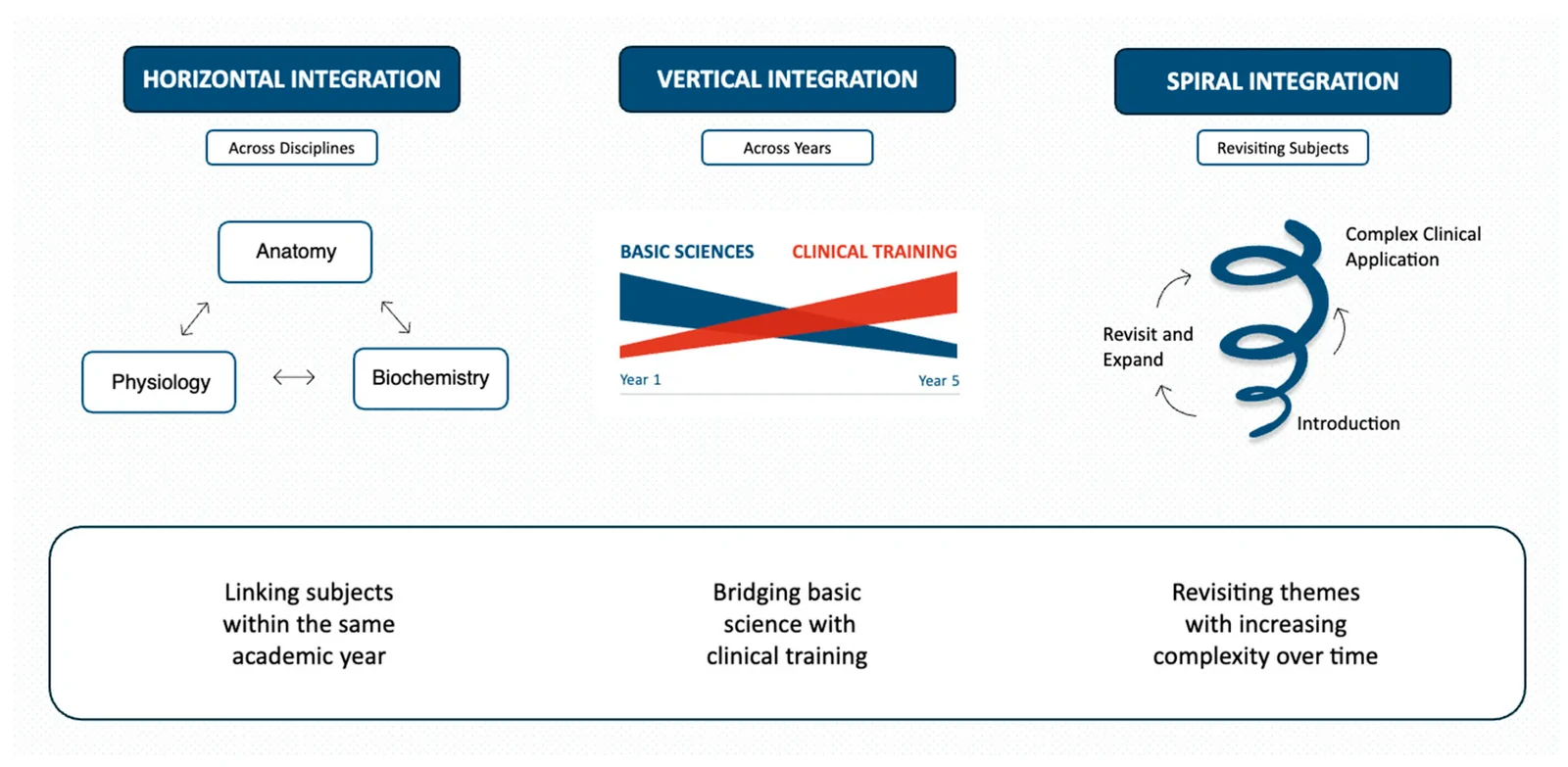
Conceptual illustration of the three main models of curricular integration: Horizontal, Vertical and Spiral.

Several models of curriculum integration have been described in the literature. A systematic review by Allouch et al. (2024) [23] identifies three main forms of integration: horizontal, vertical and spiral Figure 1. Horizontal integration refers to the coordination of content across different subject areas in a defined period, such as aligning the teaching of anatomy, physiology and pathology in the same semester [23]. Vertical integration involves connecting basic sciences with clinical disciplines across different stages of the curriculum, allowing students to revisit basic sciences as they advance toward clinical practice [12,22]. In vertically integrated curricula, classroom-based teaching typically decreases over time as clinical exposure increases [12]. Spiral integration combines both horizontal and vertical integration, with key concepts revisited repeatedly over time at increasing levels of complexity and clinical relevance [22,23].

When assessing the value of curriculum integration, it is useful to consider evidence beyond dentistry, including findings from other health professions. Studies in pharmacy education have shown that students perceive horizontal integration as highly beneficial and supportive of meaningful learning. For example, one study of pharmacy students found that horizontally integrated teaching improved understanding of the relationships between subjects and encouraged active learning [24]. However, horizontal integration may also pose challenges, such as increased demands for coordination among faculty and the risk of fragmented learning if the integration is not sufficiently aligned [11].

Vertical integration is currently one of the most widely used forms of curriculum integration in dental education. Its main advantage is that it bridges the gap between theory and practice, enabling students to recognise the clinical relevance of basic sciences early in their education. However, vertical integration can also lead to curriculum overcrowding and requires close collaboration between basic science and clinical faculty, which can be difficult to sustain [23].

Spiral integration combines the strengths of both horizontal and vertical models by reinforcing learning through repeated exposure to core concepts over time and across disciplines [23]. This approach is closely aligned with constructivist learning theory, which suggests that knowledge is most effectively acquired when learners actively build upon prior understanding [22]. Although it is widely considered an ideal model, spiral integration is difficult to implement and requires careful curriculum planning, long-term coordination and strong institutional support [23].

## 4. Approaches to Dental Education in the Nordic Countries

Although dental education across the Nordic region is based on shared welfare-based values, the structure and duration of the programs vary. Most countries follow a five-year integrated undergraduate model. However, Iceland and Finland are notable exceptions, with programs lasting six years and five and a half years, respectively [25,26]. Beyond these differences in duration, the specific pedagogical strategies used to bridge the gap between basic science and clinical practice vary between universities.

### 4.1. Sweden

Sweden has been a pioneer in this area, particularly through the implementation of the Malmö model in the 1990s [27]. This model is characterised by its systematic use of PBL, in which students at Malmö University use clinical cases to guide their learning from the very beginning of the course. This approach is intended to help students recognise the immediate clinical relevance of basic science [27]. However, although this model is known to strengthen clinical reasoning, research indicates that it places considerable demands on faculty and requires substantial institutional coordination to prevent students from feeling overwhelmed by the responsibility of self-directed learning [28]. In retrospect, this model has undergone two revisions, most recently in 2022. Although the new model shares similarities with the original PBL model, it now places greater emphasis on challenge-based learning and contemporary pedagogical research. This allows students to engage intentionally in the active development of skills such as problem-solving and decision-making without feeling overwhelmed by an excessive sense of self-responsibility [29].

Another example of how Swedish dental education is organised can be seen at Karolinska Institutet. According to the program director for the undergraduate dental program, Nikolaos Christidis (personal communication, 23 February 2026), students are introduced to patient contact relatively early, with their first direct patient encounters typically occurring during the second year in radiology. In addition, students begin practising in the in-house dental clinic from the third semester. The program also emphasises interprofessional collaboration as an important part of the students’ education.

The curriculum appears to follow a spiral integration model, in which several core subjects reappear in later semesters at a more advanced level, such as advanced prosthodontics or advanced dental radiology.

In addition, the program includes components designed to enhance students’ professional development and sense of responsibility in clinical learning environments. For example, students in semester ten may act as supervisors for junior students during clinical courses. Beginning in the autumn semester of 2026, the program will also introduce workplace-based education (VFU) in regional healthcare settings during semesters nine and ten. The goal is to further strengthen students’ clinical experience and support their transition into professional practice. Through these placements, students will gain experience with a wider range of patient cases, develop clinical pace and efficiency in real healthcare settings and work more independently under the supervision of experienced clinicians. The initiative was introduced partly in response to stakeholder feedback indicating that newly graduated dentists often have strong theoretical knowledge but require additional time in practice to build clinical confidence, routine and efficiency in certain treatment procedures.

### 4.2. Finland

Dental education in Finland differs from that in other Nordic countries in both its duration and its emphasis on early clinical exposure. The program lasts five and a half years, with the final six months usually devoted to clinical practice in the public dental health service, where students work with a high level of responsibility under supervision. Traditionally, the curriculum has been divided into a preclinical phase followed by clinical training, but over time, there has been a shift toward closer integration of basic sciences and clinical dentistry [30].

According to Head of Dental Undergraduate Education Jaana Hannele Rautava and University Lecturer Terhi Karaharju-Suvanto at the University of Helsinki (personal communication, 16 March 2026), the program remains clearly structured into approximately two preclinical years followed by three and a half clinical years. In recent decades, however, there has been a strong effort to improve integration and continuously revise the curriculum, with formal curriculum evaluations performed approximately every four years. These revisions are intended to strengthen the connection between theoretical knowledge and clinical application.

During the preclinical phase, students are introduced to dentistry early through several structured initiatives. Simulation training begins in the first and second years, allowing students to develop manual skills in a controlled environment. In addition, students gain early clinical insight through a one-day observational placement with an orthodontist in their first year. They may also assist senior students, further supporting their understanding of clinical workflows and professional responsibilities.

The curriculum also includes introductory dental-specific courses such as “Face, Mouth and Teeth”, designed to place the basic sciences in a dental context. Infection control and basic clinical procedures are introduced early, along with courses focused on manual skills, such as dental handcraft training. After completing this training, students must pass an examination before advancing to the third year. This marks a critical transition point in the curriculum, ensuring that students have developed the technical foundation required for clinical training while also serving as an early screening mechanism for those less suited to the manual demands of the profession. Together, these elements reflect a model of vertical integration in which clinical relevance is gradually introduced during the early years of the program.

Despite these efforts, students continue to report that the transition from preclinical to clinical training can be stressful. However, early exposure to clinical environments, simulation training and assisting roles appear to reduce uncertainty and improve preparedness. Before entering the clinic and meeting their first patients in the third year, students must also complete preparatory assessments, including a mini-Objective Structured Clinical Examination (mini-OSCE), which helps ensure they are ready for patient care.

Students generally value the preclinical sciences. However, they often perceive dental-specific subjects as more directly relevant to their future profession. This is evident in the context of shared teaching with medical students. A study from Helsinki shows that dental students may struggle to stay motivated in joint basic science courses if the content is not clearly connected to dental applications. Even with PBL, engagement remains limited unless both medical and dental perspectives are meaningfully integrated. When PBL cases include both perspectives, participation improves, and students report a stronger sense of relevance and equal contribution [31].

Earlier studies from the University of Oulu showed that a major curriculum renewal was performed in the early 2000s [32]. This was in response to concerns that the previous departmental structure lacked integration and led to an overloaded program. A detailed analysis of competence requirements led to a redesign in which courses were combined into ten broader Dental Teaching Strands (DTSs), bringing together content that had previously been taught separately by different departments. This restructuring reduced overlap and the overall workload and was associated with improvements in students’ scientific reasoning and learning outcomes, as the curriculum shifted toward more authentic learning and more comprehensive patient care rather than fragmented, discipline-specific instruction [32].

### 4.3. Norway

Norway presents an interesting case study for comparing pedagogical models because the country’s three educational institutions in Oslo, Bergen and Tromsø operate under the same national curriculum while showing considerable differences in curriculum design [33]. Whereas the University of Oslo (UiO) has traditionally maintained a clearer distinction between preclinical basic sciences and clinical training, the University of Bergen (UiB) and UiT The Arctic University of Norway (Tromsø) have implemented models in which basic sciences and clinical practice are integrated much earlier into the curriculum [34]. UiT is especially distinguished by the “Tromsø Model”, which places strong emphasis on decentralised clinical rotations in rural areas [35]. This model provides a setting for testing the relevance of basic sciences when encountering a complex patient population. Reports on this decentralised model suggest that early and extensive clinical exposure contributes to greater professional maturity and clinical confidence [36].

The University of Bergen (UiB) revised its dental curriculum in 2015 to better align with modern dentistry and provide students with a more balanced workload. A key reason for the revision was that students were finding it difficult to stay motivated during the early preclinical courses. They also reported that it was hard to understand how basic science related to real-world dental practice [37]. The purpose of the new curriculum was to help students develop a strong foundation in the core dental disciplines during the first three years of study and to support the transition to more advanced, theme-based learning in the final two years of the program. The new design also expands the student thesis and introduces it earlier. In addition, the curriculum places greater emphasis on patient-centred care, meaning that the patient’s overall well-being guides clinical practice [37].

What truly distinguishes Bergen from the University of Oslo (UiO), however, is how quickly students gain hands-on experience. At UiB, students take an assisting course in their very first semester and assist senior students in the second. They begin practising on phantom heads by the fourth semester and meet their first clinic patients in the fifth [38]. In contrast, dental students in Oslo do not start working with phantom heads until their fifth semester. Their first patient contact also occurs late in the fifth semester, but only in radiology, and they do not begin regular clinical work until their sixth semester [39].

Nevertheless, research across all three Norwegian institutions indicates that students report high levels of stress and a heavy workload, regardless of when clinical integration begins [34]. This raises questions about curriculum design: although early integration of basic sciences into clinical settings may strengthen professional identity and ease the transition into professional life, it does not seem to reduce the inherent cognitive and emotional pressures of dental education. The findings suggest that the challenge for future dental education lies not only in the timing of clinical introduction but also in how integration is structured to support both knowledge retention and student well-being.

### 4.4. Denmark

In Denmark, the structure and development of the dental curriculum have undergone considerable changes in recent years. A notable example is Aarhus Universitet, which introduced a revised dental curriculum in 2022 [40]. This reform was implemented in response to concerns that the previous program had become too dense and fragmented, reducing students’ opportunities for deeper academic engagement and reflection [41].

Therefore, the new curriculum was designed with stronger pedagogical coherence and greater flexibility. By reducing content overload and eliminating unnecessary repetition across courses, the program aims to create more room for in-depth learning while remaining adaptable to future developments in dentistry and healthcare [41]. According to Associate Professor and Head of Studies Irene Dige at Aarhus University (personal communication, 3 March 2026), Aarhus has also aimed to strengthen the integration of basic sciences with clinical teaching. However, for practical reasons, full integration has not always been possible, and some basic science subjects could not be directly incorporated into clinical courses. In such cases, clinical perspectives have been incorporated into the basic science courses to keep them relevant for students.

A core principle of the revised structure is a clear patient-centred approach. Students now meet their first patients in the third semester, integrating clinical experience earlier into their education. At the same time, interdisciplinary collaboration has been strengthened. Dental students participate in shared learning activities with dental technicians and dental hygienists, reflecting the collaborative nature of modern oral healthcare. The introduction of a simulation laboratory further supports the development of clinical and technical skills in a controlled learning environment [41].

Although most core subjects have been retained, some adjustments reflect changing clinical realities. Implant placement is no longer performed on patients during undergraduate training and is instead practised only in simulation settings. In addition, full prosthodontics has received less emphasis, in line with the decreasing number of patients receiving such treatments. Digitalisation is another key priority in the updated program. Students are introduced to emerging technologies, including the use of artificial intelligence in radiology and diagnostic imaging, ensuring that graduates are better prepared for a rapidly evolving professional landscape [41].

This reform reflects broader trends in Danish dental education toward student-centred learning. For example, a study of oral microbiology courses at Københavns Universitet found that curriculum changes emphasising small-group, interactive learning increased student responsibility but initially met with resistance. Both students and teachers had to adapt to their new roles, underscoring that successful reform requires careful preparation, clear expectations and support for students as they move from passive learning to active, self-directed engagement [42].

Overall, the curriculum reform at Aarhus Universitet reflects a shift toward a more streamlined, flexible and forward-looking model of dental education in Denmark, while experiences from Københavns Universitet highlight that pedagogical innovation must be supported by guidance and assistance to ensure successful implementation.

## 5. Approaches to Dental Education in North America

Dental education in the United States and Canada differs from many European models in that applicants must complete a bachelor’s degree before being admitted to dental school [18]. The professional dental program itself typically lasts four years, making the overall dental education pathway approximately seven to eight years long. Upon completion, graduates receive either a Doctor of Dental Surgery (DDS) or a Doctor of Dental Medicine (DMD) degree, which are considered equivalent [18,43]. In addition, all graduates must obtain licensure by passing the National Board Dental Examination (Parts I and II) and a regional board licensing examination before entering clinical practice [43].

Traditionally, dental curricula in North America have been organised into two preclinical years followed by two clinical years. During the first two years, students mainly focus on basic sciences such as anatomy, physiology, biochemistry and pathology, often alongside medical students. The final two years emphasise clinical training, during which students apply their knowledge in patient care settings [18,43]. However, research examining student perspectives across U.S. and Canadian dental schools has revealed dissatisfaction with this traditional structure [44]. Students often report that the preclinical years lack clinical relevance and that there is a considerable gap between theoretical knowledge and clinical practice. Many feel insufficiently prepared for the transition into clinical practice. Interestingly, the same body of research suggests that recently graduated dentists tend to view their preclinical education more positively in retrospect, emphasising that the basic science knowledge gained during those early years is essential for understanding patients’ overall health [44]. This contrast underscores the importance of the basic sciences while also highlighting the need for better integration to increase perceived relevance and student motivation.

Despite broad agreement that the basic sciences are more important than ever in modern dental education, a U.S. study has shown a considerable decline in the number of faculty members teaching these subjects, with reductions approaching 50 per cent over recent decades [45]. This trend is especially concerning given the increasing complexity of patient care, driven by ageing populations and the growing prevalence of systemic diseases with oral manifestations. The shortage of basic science faculty raises concerns about the sustainability and quality of dental education, particularly as student enrolment continues to rise [45].

In response to these challenges, many dental schools have started shifting toward more integrated curricular models. Although a substantial proportion of institutions still teach basic and clinical sciences separately, there has been a marked increase in the adoption of integrated approaches. Educational strategies such as PBL, CBL and interprofessional education (IPE) are being used increasingly to bridge theory and practice [46,47]. These approaches are intended to strengthen critical thinking and support the application of scientific knowledge to clinical decision-making. However, implementing such models is resource-intensive and requires substantial institutional commitment, including faculty development and curriculum restructuring.

### 5.1. United States

The University of Maryland School of Dentistry (UMSOD) exemplifies these systemic trends through its implementation of the spiral curriculum model [48]. From the first year, students are introduced to a fusion of basic and clinical sciences, as demonstrated by the five-month “Operative Dentistry” course, which establishes a strong foundation in tooth morphology and biomechanical principles. By incorporating subjects such as oral microbiology and biomaterials into the early curriculum, the school effectively narrows the gap between preclinical theory and clinical application [48]. Furthermore, students encounter their first patients in the first year, an approach that ensures early clinical immersion. The institution describes itself as both patient-centred and student-centred, using innovative educational methods, including digital learning platforms and online activities [49]. However, sustaining such a highly integrated educational structure poses considerable challenges at both the institutional and student levels. According to research on U.S. dental education models, the success of an integrated curriculum depends heavily on close faculty coordination to prevent the emergence of departmental “silos”, in which courses are well organised internally but lack connection to the broader curriculum [46]. Without ongoing communication between basic science researchers and clinical instructors, the intended integration of knowledge can falter. In addition to the academic rigour, students must also manage logistical challenges specific to the Maryland model, such as mandatory off-campus rotations that require them to arrange their own transportation to external clinical sites [49]. Despite these obstacles, the model’s effectiveness is evident in UMSOD’s outcomes, with graduates consistently achieving nearly 100% pass rates on the Integrated National Board Dental Examination (INBDE) [50].

At Harvard School of Dental Medicine (HSDM), the DMD curriculum is distinguished by its deep integration with medical education, ensuring that dental students build a strong scientific foundation for clinical practice [51]. In the first year, dental students take basic science courses alongside medical students at Harvard Medical School. A key element of this period is the “Practice of Medicine” course, which introduces students to primary care and highlights the essential connection between oral and systemic health. This interdisciplinary approach is designed to transform patient care by emphasising integrated health. From the first year, students are also expected to begin a research project, underscoring the importance of scientific inquiry and evidence-based practice [51].

The curriculum uses term-based learning and CBL, incorporating methods such as flipped classrooms and early clinical exposure to foster critical thinking [51]. This is exemplified by initiatives such as the Teaming and Integrating for Smiles and Health (TISH) project, which promotes bidirectional referrals and practical screenings, such as checking for hypertension in dental settings [52]. This further underscores the importance of having a strong foundation in the basic sciences, thoroughly integrated throughout the program. In the second year, the focus shifts toward the *Oral Systems Framework*, covering specialised subjects such as head and neck anatomy, oral microbiology and radiology. During this time, students also participate in the Emergent Clinical Experience, rotating through hospital-based and in-house dental clinics while observing faculty and residents. This phase marks their transition to treating their own patients. The third and fourth years are devoted to intensive clinical training, during which students apply the comprehensive basic science and scientific knowledge acquired in their first two years to direct patient care [51].

### 5.2. Canada

The University of Toronto (UofT) Faculty of Dentistry follows a curriculum design based on the Association of Canadian Faculties of Dentistry (ACFD) framework. This model requires that a competent entry-level dentist successfully integrate knowledge, skills and values across the basic, behavioural and clinical sciences [53]. Following the standard North American four-year model, the curriculum emphasises the basic sciences in the first two years while using pedagogical methods such as CBL, seminars and simulation to foster critical thinking [53,54]. According to their self-study report, evidence-based dentistry serves as the underlying principle for all clinical teaching [53].

The DDS program has evolved continuously under successive strategic frameworks. A considerable and well-documented period of change was driven by the 2014–2019 Strategic Plan, which launched major curricular reforms to strengthen both vertical and horizontal integration across the program. This progress continued under the 2019–2022 Strategic Plan, which focused on further refining the DDS curriculum [55]. A key priority during this more recent phase has been comprehensive curriculum mapping to identify and address academic gaps and redundancies, ensuring that research and scientific discovery are directly integrated into clinical pedagogy [55].

A major component of the faculty’s integrative approach is early clinical exposure. During their first and second years, students participate in intramural rotations, assisting senior students and allowing them to observe the clinical application of theory early on [53]. Additionally, extramural rotations at major hospitals, such as Mount Sinai, provide students with essential experience in treating medically compromised and disabled patients. The success of these curricular structures is reflected in student outcomes. Data from the 2022 Self-Study show that graduates typically achieve a cumulative grade point average (GPA, a 0–4.0 academic grading scale used in North America) between 3.42 and 3.62, with a seven-year graduation rate of 98.5% [53]. These results indicate that the program requirements are well aligned with student capabilities.

Despite these positive outcomes, the Faculty identifies several continuing challenges in its 2022 Self-Study [53]. Financial sustainability and the recruitment of full-time academic clinicians remain major obstacles, particularly in clinical fields [53]. To address these issues, the Faculty has implemented targeted staffing strategies, such as reducing reliance on a small percent of part-time appointments (below 40%) while gradually increasing the proportion of teaching stream faculty to better support clinical education. In addition, to strengthen communication between basic science and clinical researchers, the Faculty established monthly Research Rounds to ensure that basic science remains a central pillar of the educational experience [53].

The DMD program at the University of British Columbia (UBC) has undergone continuous change. Since 1997, dental students have taken the same basic science courses as medical students. However, in 2010, the Commission on Dental Accreditation of Canada (CDAC) recommended a formal review of this model, citing an excessive amount of basic science content that was not directly relevant to the competencies required of a dentist [56].

This prompted the faculty to perform a study using the Delphi method, involving several experts, including basic scientists, senior students and practitioners. They evaluated more than 1500 learning objectives and found that only 47% of the existing basic science courses were considered important for a dentist. While basic science topics such as human biology and cardiovascular systems remained high priorities, others, such as reproduction, were found to have only 5% relevance. Therefore, the faculty removed some of the “non-relevant” basic science courses and assumed direct control over the basic science courses themselves, rather than leaving responsibility to the medical faculty. This ensured that the basic sciences were streamlined and integrated into clinical training [56].

The curriculum used at UBC is characterised by an inquiry-based model that uses several methods, including hybrid CBL and PBL. This shift reduces reliance on traditional lectures in favour of self-directed learning. Another important feature used by the university is the inclusion of clinical groups with students from the 2nd, 3rd and 4th years. This fosters a peer-learning environment and helps younger students learn from older ones [57].

Furthermore, the faculty’s 2021–2026 Strategic Plan emphasises the need for ongoing curriculum development to adapt to an evolving professional landscape, including increased digitalisation and an ageing population. The plan also notes that students reported a need to “decompress” the study plan because they experience a high workload. In response, the strategic plan proposes assessing the academic calendar to create more space for reflection and advanced learning. By broadening access to interdisciplinary education and innovative pedagogical technologies, UBC aims to equip students to navigate complex professional practice while ensuring the long-term validity of both student and teacher assessments [58].

## 6. Approaches to Dental Education in Western Europe

### 6.1. United Kingdom

In the United Kingdom, dental education is typically organised as a five-year Bachelor of Dental Surgery (BDS) program, with a strong emphasis on integrating basic sciences and clinical practice. Compared with some other educational systems, UK dental schools place considerable focus on ensuring that the basic sciences are taught in a clinically relevant context. This is accomplished through the close alignment of theoretical instruction with patient care, as well as through early clinical exposure. Students are often introduced to patient contact in the first weeks of the program and are gradually given greater responsibility, reflecting a pedagogical approach that encourages early engagement with clinical practice [59].

At King’s College London, the BDS program has recently undergone curriculum reform, with a new curriculum introduced in the 2025/26 academic year. This updated program aligns with the General Dental Council (GDC) Safe Practitioner framework and further strengthens the integration of scientific knowledge and clinical training. A key feature of the curriculum is its spiral integration model, in which core topics are revisited with increasing complexity throughout the program. For example, students are first introduced to fundamental clinical techniques and dental materials in simulated environments before revisiting these areas in later years with a stronger emphasis on advanced procedures and clinical application. Modules such as *Simulated Clinical Skills and Dental Materials* are therefore not limited to a single stage of the program but reappear in increasingly complex forms, supporting the development of both competence and confidence [60].

In the early years, students build a strong foundation in the basic sciences, public health and the principles of clinical practice, while also being introduced to professionalism and evidence-based dentistry. This is followed by increasingly more advanced simulation-based training, which prepares them for direct patient care. From the third year onward, there is a clear transition to more intensive clinical training, with an emphasis on patient-centred care and the application of theoretical knowledge in real clinical settings. In the final years, students further refine their clinical skills, manage increasingly complex cases and prepare for independent professional practice, ensuring that all GDC learning outcomes are achieved [60].

A comparable integrated, competency-based approach is observed at Queen Mary University of London [61], where the five-year BDS program is organised around four longitudinal domains: clinical knowledge and skills, interpersonal skills, professionalism and self-management. The curriculum has recently been reviewed and updated in line with the GDC’s Safe Practitioner framework, ensuring that graduates meet current professional standards [61].

In the first year at Queen Mary, students are introduced to basic sciences, clinical principles and professional concepts, with particular emphasis on the relationship between systemic and oral health. Teaching covers subjects such as oral biology, dental materials science and the effects of physiological systems and disease on oral health. Early clinical exposure is provided through patient observation and clinical skills training, and the program encourages IPE through collaboration with dental hygiene and therapy students. The curriculum follows the same spiral integration model as King’s College [61].

During the second and third years, students increasingly apply their knowledge in clinical settings, treating patients while continuing their academic development through seminars, laboratory work and self-directed learning. The curriculum encompasses a broad range of dental disciplines, including restorative dentistry, prosthodontics, endodontics, oral medicine, oral surgery and orthodontics. Clinical training is further strengthened through placements in outreach centres, where students gain experience in treating patients in community settings and develop skills in prevention and treatment planning [61].

In the fourth and fifth years, the emphasis shifts to consolidating clinical competence and preparing for independent practice. Students encounter a broader and more complex range of cases, including hospital-based treatments and are encouraged to assume greater responsibility in patient care. In addition, they have the opportunity to explore areas of personal interest through student-selected modules, which may include research, quality improvement projects and specialised clinical or theoretical subjects [61].

Overall, dental education in the United Kingdom is characterised by a well-structured integration of basic sciences and clinical training, early and ongoing patient contact and the use of spiral curricula to reinforce learning over time. National frameworks such as the GDC’s Safe Practitioner model further ensure consistency and alignment with the competencies required for safe and effective clinical practice. Notably, several programs have recently undergone curricular revisions, indicating an ongoing effort to modernise dental education. This ongoing development indicates that UK dental curricula are adapting to evolving educational standards, technological advances and the changing demands on dental professionals, thus better equipping them to address contemporary challenges in dental education and practice.

### 6.2. Spain

The dental program at Universitat Internacional de Catalunya in Barcelona is a five-year undergraduate degree designed as a gradual progression from basic sciences to clinical practice. According to Dr. María Arregui Gambús (personal communication, 1 May 2025), the first year, as well as part of the second, is primarily dedicated to basic sciences, ethics and anthropological principles. Important core subjects include Human Physiology, Anatomy, Histology and Microbiology. These subjects provide the basis for the clinical training undertaken in the later years.

In the second year, dental-specific content is introduced alongside training in simulation laboratories that use both physical models and digital tools. Clinical orientation also begins that year, with students spending 30 h observing and shadowing final-year students in clinical settings. In the second semester of the second year, students complete 15 clinical hours through the *Preventive Dentistry 2* course, performing basic preventive procedures on fellow students and monitored patients. Throughout the third year, simulation training is supplemented by four hours of clinical training each week. Clinical progression depends on each student attaining an adequate level of practical skill, rather than adhering to a fixed academic timeline. In the fourth and fifth years, direct patient care becomes the primary mode of learning as preclinical training is gradually phased out.

The curriculum follows a vertically integrated model, in which basic science content reappears in specialised clinical subjects. Students are expected to apply their earlier knowledge of microbiology and histology when engaging with the pathological mechanisms underlying conditions treated in endodontics and periodontics. Students generally report recognising this connection in their clinical work, particularly in areas that require anatomical knowledge, such as tooth restoration and the administration of local anaesthesia.

Overall, students generally feel prepared when entering clinical training, although confidence differs across treatment areas. Higher confidence is usually reported in restorative and periodontal procedures, whereas prosthodontics tends to be more challenging. In response, the faculty adjusts the amount of preclinical preparation based on each student’s individual development, ensuring that readiness, rather than a fixed schedule, determines the transition to patient care.

Curriculum quality is monitored through multiple mechanisms, including annual reports, periodic internal assessments of teaching staff and external reviews by the Ministry of Education performed approximately every five years. The program has also been evaluated through the ADEE LEADER project, a European peer review system for quality assurance in dental education [62], where it received a favourable assessment. In terms of curriculum development, the program underwent a major restructuring in 2013/2014 to align with the Bologna Process and is currently being updated in response to revised Spanish legislation and the latest European directive on professional qualifications. These ongoing revisions reflect a broader institutional commitment to ensuring the program remains aligned with contemporary educational standards, digital developments in dentistry and evolving competency requirements across Europe.

Although the program at Universitat Internacional de Catalunya represents one approach to dental education in Spain, characterised by early clinical integration and individualised progression, a different model can be found at the Complutense University of Madrid.

According to Professor David Herrera González (personal communication, 17 April 2026), the dentistry program at the Complutense University of Madrid is a five-year undergraduate degree structured in accordance with European directives and Spanish legislation. Basic sciences represent 25% of the total curriculum and are concentrated primarily in the first two years. They cover a broad range of subjects, including anatomy, physiology, biochemistry, microbiology, oral biology, public health and psychology, providing students with a scientific foundation that spans biological, behavioural and population-based perspectives.

The program follows a largely sequential structure, with basic science content taught before, rather than alongside, clinical training. Early vertical integration is limited, and although some clinically oriented electives attempt to bridge this gap in later years, they remain relatively isolated. Intensive clinical exposure does not begin until the fourth year, while comprehensive patient care is introduced in the fifth year. This delayed transition has been associated with lower student confidence and increased stress when students first encounter patient-based settings. Students often perceive the basic sciences as highly theoretical, with their clinical relevance becoming apparent only in the later stages of the program.

Although the program’s strong foundation in the basic sciences and substantial clinical training in the final years are recognised as key strengths, the curriculum has been critically assessed as having limited interdisciplinary approaches and insufficient early integration between the basic and clinical sciences. The curriculum also provides insufficient coverage of certain foundational areas, such as microbiology and immunology. In addition, the incorporation of digital dentistry and transversal competencies has been identified as requiring further development. No formal published studies evaluating the current curriculum are available, and assessment relies primarily on institutional quality assurance mechanisms and student feedback. Therefore, a revised curriculum is planned for introduction in the 2026–2027 academic year, driven by Spanish Royal Decree 822/2021 and updated European directives. The reform aims to advance a more competency-based and student-centred model, with stronger integration of scientific and clinical content throughout the program, enhanced CBE, expanded use of clinical cases in early instruction and broader incorporation of digital technologies in dental practice.

### 6.3. Germany

In Germany, dental education has been regulated by national licensing rules since 1955, requiring a strict separation between five preclinical and five clinical semesters. Unlike the medical curriculum, which has been revised multiple times over the same period, these regulations have remained largely unchanged, formally preventing the early integration of clinical content into preclinical training and limiting the adoption of modern teaching approaches [63].

Despite these national regulations, 29 of the 30 dental schools in Germany had launched local reform initiatives by 2011. The most common reform, implemented at 16 institutions, involved combining previously separate clinical courses in restorative dentistry and prosthodontics into integrated treatment programs. At 11 institutions, clinical perspectives were intentionally incorporated into the preclinical phase through approaches such as early patient contact, clinical observations and dental practice placements. In addition, eight institutions had introduced Objective Structured Clinical Examinations (OSCEs), five had adopted problem-based learning, and five had implemented e-learning or blended-learning formats. An additional 18 institutions offered elective course series on topics such as oral physiology, communication skills and psychology in dentistry, reflecting an effort to expand dental education beyond its traditional technical focus [63].

Several institutions stand out as especially innovative. At the University of Greifswald, a structured early patient contact program was introduced across the first four semesters of the preclinical phase. Guided by a Community Dentistry approach, students visited patients in nursing homes and addiction clinics, performed structured medical histories and developed individualised preventive programs. This initiative was designed to promote an early understanding of the relationship between oral and general health and concluded with an OSCE examination using standardised patients. At the University of Düsseldorf, a dedicated oral physiology course was developed as part of a broader model curriculum. Building on the general physiology content shared with medical students, the course enhanced students’ understanding of orofacial physiological processes and introduced pathophysiological conditions relevant to dental practice, thus connecting basic science instruction with clinical application. At the University of Heidelberg, the HeiCuDent curriculum reorganised the entire program into three phases, eliminating strict disciplinary boundaries in favour of interdisciplinary simulation and treatment courses in which the patient, rather than the individual subject, became the central focus of teaching [63].

Overall, Germany’s experience shows how innovation at the institutional level can partly offset structural national regulations. However, the authors of the reviewed study emphasise that sustainable and widespread reform requires updated national regulations that provide sufficient flexibility for institution-specific profiles while ensuring adequate resources, appropriate faculty-to-student ratios and greater compatibility between dental and medical education.

## 7. Approaches to Dental Education Outside the Western World

### 7.1. China

Dental education in China is characterised by a complex, multi-tiered structure with three main educational pathways: five-, seven- and eight-year programs. These lead to different qualifications, namely a Bachelor of Dental Surgery (BDS), Master of Stomatological Medicine (SMM) and Doctor of Stomatological Medicine (SMD), respectively. The only requirement for becoming a dental student is achieving a certain score on the College Entrance Examination (CEE) [59].

The various dental education pathways have created confusion for students. To address this, China has adopted a 5 + 3-year model as its primary program. In this system, most students complete a 5-year dental degree followed by a 3-year residency to build clinical competence. Therefore, the 7-year program was discontinued in 2015 [59].

China’s undergraduate curriculum largely follows a traditional, discipline-based structure. At institutions such as the West China School of Stomatology, the first two years are primarily dedicated to basic science subjects. During the third year and the first half of the fourth year, students are introduced to clinical medicine as well as foundational dental subjects, including oral histology and pathology and oral anatomy and physiology. The second half of the fourth year then shifts its focus to dental clinical and experimental courses. In the final year, students complete clinical rotations in major departments, including oral medicine, oral and maxillofacial surgery, prosthodontics and orthodontics, typically at affiliated teaching hospitals [59].

The curriculum is structured to place strong emphasis on theoretical and general academic subjects during the early years of study, while clinical training is largely compressed into the final year of the program. This creates an intensive transition from predominantly classroom-based learning to full-time clinical practice. In response to concerns about limited clinical contextualization in the early phases, some institutions have introduced introductory dental courses, as well as subjects such as psychology and aesthetics, to enhance student engagement and provide earlier professional orientation. Some schools have also explored PBL/CBL methods. Nevertheless, the overall structure continues to reflect a clear separation between the basic sciences and clinical training [59].

Preclinical training is supported by simulation laboratories in many dental schools, but access to equipment and practice time may be limited, particularly as student enrolment increases. This can restrict the development of technical skills before students enter clinical settings. Clinical training itself is typically intensive, with students exposed to a high volume of patients during their final year. While this provides valuable hands-on experience, the heavy workload may reduce opportunities for reflection and the integration of knowledge [59].

Postgraduate training plays a crucial role in the transition from student to independent practitioner. Since the formal introduction of standardised residency programs in 2015, clinical competence has increasingly been developed during this phase rather than during undergraduate education. Residency programs include rotations across multiple dental specialities, followed by optional specialisation pathways. In addition, licensure requires passing both clinical and theoretical examinations after graduation, further underscoring the importance of postgraduate training [59].

Overall, dental education in China reflects a system undergoing transition. While recent reforms aim to strengthen clinical competence and standardise training, the curriculum remains largely discipline-based, with limited early integration of the basic and clinical sciences. The increasing adoption of student-centred learning methods and curricular adjustments suggests a gradual shift toward more integrated and flexible educational models, although considerable variation persists among institutions.

### 7.2. Nigeria and South Africa

Dental education in Africa faces unique challenges shaped by the continent’s distinct disease burden and healthcare context. In many African countries, dentists are expected to manage a wide range of general health conditions in addition to oral health, making a strong foundation in the basic sciences especially important. This places greater demands on dental curricula to ensure that the basic sciences are meaningfully integrated into clinical training. The following examples from Nigeria and South Africa illustrate how some institutions have addressed this challenge through considerable curriculum reform.

At the University of Ibadan in Nigeria, they shifted from traditional, siloed models to Competency-Based Medical Education (CBME) [64]. Over a five-year period, from 2005 to 2010, the institution transitioned from a 60-year-old traditional curriculum to an integrated, student-centred approach to bridge the “disconnect” students often felt between preclinical theory and clinical practice. To ensure the curriculum reflected the modern world, the school performed an extensive review process involving surveys, interviews and discussions with various stakeholders. The consensus was clear: despite its long history, the old curriculum was no longer producing graduates able to meet the country’s current and future health needs. A critical part of this process involved identifying the strengths and weaknesses of the traditional system. While the old model provided a strong theoretical foundation and benefited from experienced staff, it had several shortcomings, such as a lack of integration and insufficient understanding of the importance of basic science [64].

The new curriculum uses horizontal integration in the first two semesters, during which subjects such as Anatomy, Physiology and Biochemistry are harmonised and taught thematically. In the third semester, vertical integration is introduced through the course *Clinical Application of Basic Sciences*, in which basic scientists and clinicians co-teach to demonstrate the immediate relevance of theory to patient care [64]. The new curriculum also allowed time for new courses such as *medicine/dentistry as a profession* and *multidisciplinary healthcare delivery*, which involve teams of students from other health professions to give students an early introduction to how a team-based approach in health care works [64].

To ensure depth of learning without overwhelming students, the Ibadan model uses a prioritization strategy known as the 50/30/10 Rule. Curriculum content is categorized into Must Know (50% of instruction time), Should Know (30%) and May Know (10%). To maintain high standards and avoid the PBL trap, in which students may gain only surface-level knowledge, the school uses a non-compensatory assessment method. This requires students to pass each individual basic science subject and achieve a minimum score in the Must Know section to progress [64].

Despite the immense effort invested in these curriculum reviews, the transition was challenging and encountered considerable resistance. This included resistance from faculty members who believed that a doctor must know everything, which made the new prioritization model difficult to accept. Older faculty members, in particular, saw no need for change, arguing that the existing plan had worked effectively for 60 years. Students also experienced anxiety, with some feeling like guinea pigs in a new system, further pressured by an increase in the pass mark from 75% to 80%. From a logistical standpoint, the school also had to address infrastructure shortcomings, such as unreliable electricity and internet access, along with a strained financial situation because developing a new curriculum and implementing modern methods and tools require substantial financial investment [64].

To address these challenges, the institution introduced ongoing faculty development and mentoring programs. Notably, students took the initiative to organise their own mock examinations and peer study groups to fill gaps where teachers struggled to adapt. The Alumni Association also played a vital role by donating resources such as buses and research centres [64]. Despite initial resistance and logistical difficulties, the reform has proven successful. Clinical teachers report that students from the new curriculum demonstrate satisfactory, or even superior, application of the basic sciences compared with those in the old model. The school has risen in international rankings and the Ibadan Model is now serving as a blueprint for other medical and dental schools across Africa and Asia [64].

The need for such reforms is further supported by findings from the University of Pretoria, where a study specifically examined how different instructional designs influence dental students’ ability to connect these disciplines [65]. To break down traditional silos, the university introduced CBL and a *Comprehensive Patient Care* course. The results showed that students who worked with real-life cases in their third year rated horizontal integration very highly, with scores reaching 80/100. A key success factor was the presence of dedicated faculty; students mentored by teachers specifically focused on integration scored 9–15% higher than those in traditional settings [65].

However, the study also indicates that students found vertical integration particularly challenging, reporting that it was considerably harder to connect new clinical knowledge with subjects they had studied in previous years. Another possible reason for the negative feedback on vertical integration is curriculum overload, in which students feel overwhelmed by a lack of clear relevance between past and present courses [65].

This article also notes that basic science disciplines, such as Microbiology and Pathology, are taught as comprehensive, standalone subjects. Because these courses are often content-heavy and delivered in full, much of the material lacks a clear or direct connection to typical dental case studies. Furthermore, poor communication between academic departments means that clinical instructors may not know exactly what was covered in previous years, making it difficult for them to help students build the necessary conceptual bridges. To address this, the study suggests strengthening vertical integration through improved interdepartmental collaboration and a deliberate reduction of unnecessary content [65].

### 7.3. Saudi Arabia

Dental education in Saudi Arabia follows a nationally regulated seven-year structure established by the Ministry of Higher Education and the National Commission for Academic Accreditation and Assessment (NCAAA). The program includes a one-year health foundation program, five years of dental education and one year of compulsory internship. Students are admitted directly after secondary education, and the foundation year provides a broad introduction to basic sciences and academic skills [66].

The dental curriculum is usually structured progressively. The first two years focus on theoretical knowledge, procedural understanding and the development of fine motor skills, followed by a gradual transition to clinical training. Beginning in the third year, students combine preclinical training with clinical exposure, while the final years emphasise comprehensive patient care alongside continued instruction in the basic and professional sciences [66].

At institutions such as Princess Nourah bint Abdulrahman University College of Dentistry (PNUCD), the curriculum is organised into “blocks” and “stream courses”. Block courses are short, sequential and skills-based, allowing students to focus intensively on specific competencies, while stream courses run longitudinally and provide theoretical support. As students progress, clinical courses increasingly integrate knowledge and skills from both components through comprehensive care models [66].

Basic sciences are integrated throughout the program. These subjects are introduced early using a system-based and thematically integrated approach, often supported by PBL. In later years, teaching focuses more explicitly on the clinical relevance of systemic diseases and their implications for dental care. Early clinical exposure is also included, beginning with observation and preventive care and later advancing to participation in supervised, vertically integrated clinical teams. In addition, IPE and simulation-based training are key components of the curriculum. Collaboration with medical students and the use of advanced simulation technologies are intended to strengthen both clinical reasoning and readiness for patient care.

Curriculum evaluations indicate generally positive student perceptions, particularly of the block structure and the use of PBL, which are associated with improved focus, critical thinking and long-term knowledge retention. Early clinical exposure is also reported to enhance confidence. However, students frequently report high levels of stress, especially in relation to the combination of block and stream courses and the limited time for independent study. Faculty feedback likewise highlights challenges related to course overlap and the need for further training in student-centred pedagogical approaches [66].

In summary, dental education in Saudi Arabia reflects a structured yet evolving model, in which efforts to integrate basic sciences and clinical practice are evident, but successful implementation still depends on balancing workload, pedagogical support and curricular coherence.

## 8. Future Directions

Although the curricula reviewed differ considerably in structure, educational philosophy and national context, several cross-cutting themes emerge consistently across countries and institutions. These include the timing and integration of basic science teaching, the role of early clinical exposure, the implementation of student-centred learning approaches, curriculum overload, the importance of faculty coordination and the limited availability of independent evaluation studies. Together, these recurring themes provide a broader perspective on the opportunities and challenges associated with curriculum integration in contemporary dental education.

This review’s findings reveal both a consensus on the importance of integrating basic sciences with clinical training and considerable variation in how this integration is implemented in practice. Despite decades of reform efforts, the traditional structure of approximately two preclinical years followed by clinical training remains dominant in many programs examined in this review. This persistence suggests that changing the structure of dental education is rarely straightforward. It is shaped not only by educational aspirations but also by national regulations, available resources and long-standing institutional traditions.

### 8.1. The Promise and Complexity of Integration

A recurring theme across the programs examined is that curriculum integration, although widely supported, is far more difficult to implement effectively than to adopt in principle. Effective integration requires sustained coordination among faculty across departments and disciplines. Without this, courses may remain internally coherent yet disconnected from the broader curriculum. These observations suggest that curriculum reform cannot be reduced to pedagogical design alone. Across the educational systems reviewed, successful integration appears to depend less on the specific curriculum model adopted than on the quality of its implementation and the extent of collaboration between basic science and clinical faculty.

### 8.2. Problem-Based Learning: Promise and Preconditions

PBL has been implemented across many of the programs reviewed, and its value in contextualising basic science is well established. However, the evidence reviewed here indicates that its effectiveness is highly dependent on how it is implemented. Shared PBL cases in interdisciplinary settings seem to motivate students only when the case design genuinely incorporates both professional perspectives. When this is not achieved, PBL may have the opposite of its intended effect, leaving students disengaged rather than motivated. In addition to case content, PBL also places substantial demands on teaching staff. Faculty must be well trained and confident in facilitating self-directed inquiry; without this investment, the method may increase student anxiety rather than cultivate independent thinking. This points to a broader principle: no matter how well designed a teaching method is, it will be only as effective as the faculty delivering it. This finding also reflects a broader pattern observed throughout the review: educational methods cannot be evaluated independently of the institutional context in which they are implemented. Similar teaching approaches may therefore produce different outcomes across institutions, despite sharing the same underlying educational philosophy.

### 8.3. Early Clinical Exposure and Assistive Roles

Across the programs reviewed, early clinical exposure consistently stands out as one of the more effective strategies for bridging the gap between theory and practice. Notably, even modest forms of early engagement, such as observation placements, simulation training and assisting senior students, seem to produce meaningful gains in student confidence and professional identity. This suggests that the benefit depends not only on the amount of early clinical contact, but also on its quality and structure. Gradually allowing students to enter clinical environments in a supported, low-pressure way appears to make the transition to independent patient care feel less overwhelming, which students across many countries identify as one of the most stressful parts of their education.

Finland’s use of a practical skills examination in the first year, followed by a mini-OSCE before initial patient contact, reflects an especially thoughtful approach to managing this transition. Rather than relying on fixed timelines, readiness is assessed and confirmed before progression, an approach that also serves as an early and humane means of identifying students who may be unsuited to the manual demands of the profession. Across the institutions reviewed, the common denominator was not necessarily how early students entered the clinic, but rather how carefully that transition was planned and supported. This suggests that the quality and educational design of early clinical experiences may matter more than their exact timing.

### 8.4. Curriculum Content: What to Include and What to Let Go

Several programs reviewed have made decisions to reduce or remove content that is no longer considered directly relevant to contemporary dental practice. This reflects a maturation in curriculum thinking: reform must not only respond to new demands by adding content, but must also be willing to let go of content that no longer serves graduates’ professional needs. This is important given consistent reports of cognitive overload and heavy workload across multiple educational contexts. Removing unnecessary overlap and outdated material creates space for deeper engagement with what truly matters and may do more to support student well-being than adjustments to integration timing alone. Considering all these observations together, curriculum reform should involve not only decisions about how knowledge is integrated, but also ongoing evaluation of which knowledge is most relevant to contemporary dental practice.

At the same time, the lasting importance of basic sciences must not be underestimated. The difference between how students perceive this content during training and how graduates value it after graduation is remarkable and points to a need not for less basic science, but for a more visible integration of its clinical relevance throughout the curriculum. The declining number of faculty teaching these subjects in some countries is therefore concerning and warrants serious institutional attention.

### 8.5. Gaps in the Evidence

The breadth and timeliness of the available literature varied considerably across regions. In several cases, the most detailed evaluations of a given curriculum were a decade old or older and more recent publications were either unavailable or non-existent. This did not reflect selective inclusion, but rather the current state of the literature. This is itself a finding: the relative lack of recent, independently published curriculum evaluations highlights a gap in the field that future research should address. Indeed, this may be one of the most important findings of this review because it limits meaningful comparisons between educational models and makes it difficult to determine which curricular approaches are truly associated with improved educational outcomes.

Many reforms rely on internal assessments, student satisfaction surveys, or licensing examination pass rates, all of which are useful but insufficient for understanding how different curricular approaches influence long-term knowledge retention, clinical reasoning, or graduate preparedness. Several curricula reviewed here have no published evaluation studies at all, and many reforms are too recent for longitudinal data to have accumulated. While this is understandable, it limits the conclusions that can be drawn. Future curriculum development should be accompanied by systematic, independent evaluation that centres students’ experience alongside outcomes data, enabling the field to build a more meaningful and comparable evidence base.

## 9. Conclusions

Across the diverse educational systems included in this review, several recurring patterns emerge despite considerable variation in curriculum structure, educational philosophy and the nature of the available evidence. Curriculum integration is widely recognised as essential for preparing dental graduates for contemporary clinical practice, yet institutions differ substantially in how this objective is implemented. Approaches vary in the timing of basic science instruction, early clinical exposure, the use of integrated teaching strategies such as PBL and the balance between traditional discipline-based teaching and longitudinal integration.

The evidence suggests that no single model is universally superior; rather, the effectiveness of any curricular approach depends on the quality of faculty coordination, the deliberateness of its design and the extent to which clinical relevance is made visible to students throughout their training.

An important observation from this review is that few institutions adhere to a single educational model in its pure form. Instead, most curricula incorporate elements from multiple integration strategies, which make it difficult to classify programs under one dominant model. This hybrid approach likely reflects the complexity of curriculum development and the need to adapt educational principles to local institutional contexts, available resources and regulatory requirements.

Without rigorous and independent evaluation, curriculum reform risks being shaped primarily by educational philosophy and institutional preference rather than by evidence of better student learning, clinical reasoning and graduate preparedness. Therefore, strengthening this evidence base should be considered a priority for future research in dental education.

## Data Availability

The original contributions presented in this study are included in the article. Further inquiries can be directed to the corresponding author.

## Supplementary Materials

The following supporting information can be downloaded at https://www.mdpi.com/article/10.3390/dj14080529/s1. Table S1. Comparative summary of dental curricula across some of the included institutions.

## References

[1] Bertolami C.N. (2001). Rationalizing the dental curriculum in light of current disease prevalence and patient demand for treatment: Form vs. content. J. Dent. Educ..

[2] Dennis M.J. (2010). Integration of medicine and basic science in dentistry: The role of oral and maxillofacial surgery in the pre-doctoral dental curriculum. Eur. J. Dent. Educ..

[3] Dovjak P. (2022). Polypharmacy in elderly people. Wien. Med. Wochenschr..

[4] Custers E. (2010). Long-term retention of basic science knowledge: A review study. Adv. Health Sci. Educ..

[5] Safarnavadeh M., Koumleh S.E., Jafari S.E.M. (2019). Desirable Status of the Dental Curriculum Based on the National Requirements of Iran and International Experiences. J. Med. Educ. Dev..

[6] Harden R.M., Sowden S., Dunn W.R. (1984). Educational strategies in curriculum development: The SPICES model. Med. Educ..

[7] Henzi D., Davis E., Jasinevicius R., Hendricson W. (2006). North American dental students’ perspectives about their clinical education. J. Dent. Educ..

[8] National Research Council, Division of Behavioral, Social Sciences and Education, Board on Behavioral, Cognitive, Sensory Sciences, Committee on Developments in the Science of Learning with additional material from the Committee on Learning Research, Educational Practice (2000). How People Learn: Brain, Mind, Experience, and School: Expanded Edition.

[9] Dornan T., Littlewood S., Margolis S.A., Scherpbier A., Spencer J., Ypinazar V. (2006). How can experience in clinical and community settings contribute to early medical education? A BEME systematic review. Med. Teach..

[10] Ghassan A., Shukr I., Sadiq N., Ahsan R. (2021). Current trends in dental education. Pak. Armed Forces Med. J..

[11] Matinho D., Pietrandrea M., Echeverria C., Helderman R., Masters M., Regan D., Shu S., Moreno R., McHugh D. (2022). A Systematic Review of Integrated Learning Definitions, Frameworks, and Practices in Recent Health Professions Education Literature. Educ. Sci..

[12] Brauer D.G., Ferguson K.J. (2015). The integrated curriculum in medical education: AMEE Guide No. 96. Med. Teach..

[13] Crawford J.M., Adami G., Johnson B.R., Knight G.W., Knoernschild K., Obrez A., Patston P.A., Punwani I., Zaki A.M., Licari F.W. (2007). Curriculum restructuring at a North American dental school: Rationale for change. J. Dent. Educ..

[14] Barrows H.S. (1996). Problem-based learning in medicine and beyond: A brief overview. New Dir. Teach. Learn..

[15] Schmidt H.G., Rikers R.M. (2007). How expertise develops in medicine: Knowledge encapsulation and illness script formation. Med. Educ..

[16] Patel V.L., Evans D.A., Kaufman D.R. (1990). Reasoning strategies and the use of biomedical knowledge by medical students. Med. Educ..

[17] De Bruin A.B.H., Schmidt H.G., Rikers R.M.J.P. (2005). The Role of Basic Science Knowledge and Clinical Knowledge in Diagnostic Reasoning: A Structural Equation Modeling Approach. Acad. Med..

[18] Licari F.W., Evans C.A. (2017). Clinical and Community-Based Education in U.S. Dental Schools. J. Dent. Educ..

[19] Manogue M., McLoughlin J., Christersson C., Delap E., Lindh C., Schoonheim-Klein M., Plasschaert A. (2011). Curriculum structure, content, learning and assessment in European undergraduate dental education—Update 2010. Eur. J. Dent. Educ..

[20] Spielman A.I. (2024). Dental education and practice: Past, present, and future trends. Front. Oral Health.

[21] Kristensen A.T., Thune N.H., Khan Q., Utheim T.P., Sehic A. (2024). The Importance of Basic Sciences in Dental Education. Dent. J..

[22] Harden R.M. (2000). The integration ladder: A tool for curriculum planning and evaluation. Med. Educ..

[23] Allouch S., Ali R.M., Al-Wattary N., Nomikos M., Abu-Hijleh M.F. (2024). Tools for measuring curriculum integration in health professions’ education: A systematic review. BMC Med. Educ..

[24] Mawdsley A., Willis S. (2019). Exploring an integrated curriculum in pharmacy: Students’ perspectives on the experienced curriculum and pedagogies supporting integrative learning. Curr. Pharm. Teach. Learn..

[25] Fiehn N.E. (2002). Perspectives on dental education in the Nordic countries. J. Dent. Educ..

[26] University of Eastern Finland (2026). Insitute of Dentistry Kuopio: University of Eastern Finland. https://www.uef.fi/en/unit/institute-of-dentistry.

[27] Rohlin M., Petersson K., Svensater G. (1998). The Malmo model: A problem-based learning curriculum in undergraduate dental education. Eur. J. Dent. Educ..

[28] Lundegren N., Jonsson A., Lindberg P. (2021). An upgrade of the Malmo model by implementing case-based teaching and learning, in an undergraduate dental education. Eur. J. Dent. Educ..

[29] Paulsson L.G., Christina, Hedenbjörk-Lager, Anders, Lindberg P., Wahlin Å., Lundeg N. (2025). Ny tandlægeuddannelse i Malmö søger at møde fremtidens behov. Tandlaegebladet.

[30] Kerosuo E., Ruotoistenmaki J., Murtomaa H. (2001). Report on the development of a new dental curriculum at Helsinki. Eur. J. Dent. Educ..

[31] Mussalo F., Karaharju-Suvanto T., Mäntylä P., Pyörälä E. (2021). Biomedical Courses Should Also Be Designed for Dental Students: The Perceptions of Dental Students. Dent. J..

[32] Hietala E.L., Karjalainen A., Raustia A. (2004). Renewal of the clinical-phase dental curriculum to promote better learning at the University of Oulu. Eur. J. Dent. Educ..

[33] Komabayashi T., Astrom A. (2007). Dental education in Norway. Eur. J. Dent. Educ..

[34] Kristensen A.T., Thune N.H., Khan Q., Utheim T.P., Hammer H.L., Sehic A. (2026). Student Perspectives on Integrating Basic and Clinical Dental Sciences: Insights from Three Dental Schools in Norway. Front. Dent. Med..

[35] Eriksen H.M.H., Arne (2009). Tromsømodellen Hva særpreger tannlegeutdanningen i Tromsø?. Den. Nor. Tann. Tid..

[36] Eriksen H.M., Bergdahl M., Byrkjeflot L.I., Crossner C.G., Widstrom E., Tillberg A. (2011). Evaluation of a dental outreach teaching programme. Eur. J. Dent. Educ..

[37] Det Medisinsk-Odontologiske Fakultet, Universitetet i Bergen (2016). Prosjektskisse for ny Studieplan—Integrert Masterprogram i Odontologi.

[38] Universitetet i Bergen Odontolo (Integrert Master) 2026. https://www4.uib.no/studier/program/odontologi-integrert-master.

[39] Universitetet i Oslo Odontologi (Master) Oppbygging og Gjennomføring 2026. https://www.uio.no/studier/program/odontologi/oppbygging/.

[40] The Eyes of the World Are Resting on the Aarhus Model of Dental Education Aarhus University 2021. https://international.au.dk/currently/news/preview/artikel/the-eyes-of-the-world-are-resting-on-the-aarhus-model-of-dental-education.

[41] Tandlægebladet (2025). Tandlægestuderende Skal Håndtere Mere Komplekse Situationer.

[42] Netterstrom I., Fiehn N.E., Larsen T. (2011). Changing the curriculum and the role of the teacher and the students in the classroom—An analysis of the process of reforming a course in oral microbiology. Eur. J. Dent. Educ..

[43] Wu Z.Y., Zhang Z.Y., Jiang X.Q., Guo L. (2010). Comparison of dental education and professional development between mainland China and North America. Eur. J. Dent. Educ..

[44] Henzi D., Davis E., Jasinevicius R., Hendricson W. (2007). In the students’ own words: What are the strengths and weaknesses of the dental school curriculum?. J. Dent. Educ..

[45] Formicola A.J. (2017). Trends in Dental Faculty of U.S. Dental Schools, 2003–04 to 2013–14. J. Dent. Educ..

[46] Elangovan S., Venugopalan S.R., Srinivasan S., Karimbux N.Y., Weistroffer P., Allareddy V. (2016). Integration of Basic-Clinical Sciences, PBL, CBL, and IPE in U.S. Dental Schools’ Curricula and a Proposed Integrated Curriculum Model for the Future. J. Dent. Educ..

[47] Haden N.K., Hendricson W.D., Kassebaum D.K., Ranney R.R., Weinstein G., Anderson E.L., Valachovic R.W. (2010). Curriculum change in dental education, 2003–2009. J. Dent. Educ..

[48] University of Maryland School of Dentistry Doctor of Dental Surgery (DDS) Curriculum 2023. https://dental-umaryland.smartcatalogiq.com/en/2023-2024/catalog/the-doctor-of-dental-surgery-dds-program.

[49] University of Maryland School of Dentistry (2026). Doctor of Dental Surgery Program (DDS).

[50] University of Maryland Baltimore (2025). Professional Licensing Exam Pass Rates Baltimore, MD: University of Mary-land, Institutional Effectiveness, Strategic Planning, and Assessment (IESPA). https://www.umaryland.edu/iespa/institutional-effectiveness/professional-licensing-exam-pass-rates/.

[51] Harvard School of Dental Medicine DMD Program 2026. https://www.hsdm.harvard.edu/dmd-program.

[52] Cardenas K., Weilnau T., Aguilar C., Ali A., Eidelman A., Ponnala S., Russel T., Schwanderla J., Sievers K., Wu H. (2023). Partnering for Integrated Care: A Learning Collaborative for Primary Care and Oral Health Teams. Ann. Fam. Med..

[53] University of Toronto Faculty of Dentistry (2022). Faculty of Dentistry Self-Study 2021.

[54] University of Toronto Faculty of Dentistry DDS Program Overview and Curriculum Delivery 2026. https://www.dentistry.utoronto.ca/prospective-students/undergraduate/DDS.

[55] University of Toronto Faculty of Dentistry (2019). Strategic Plan Update: 2019–2022.

[56] Best L., Walton J.N., Walker J., von Bergmann H. (2016). Reaching Consensus on Essential Biomedical Science Learning Objectives in a Dental Curriculum. J. Dent. Educ..

[57] University of British Columbia DMD Curriculum 2026. https://www.dentistry.ubc.ca/education/dmd/dmd-curriculum/.

[58] University of British Columbia (2026). Impact 2020–2025 Trailblazers in Oral Health for BC and Beyond.

[59] Wang Y.H., Zhao Q., Tan Z. (2017). Current differences in dental education between Chinese and Western models. Eur. J. Dent. Educ..

[60] King’s College London Dentistry BDS 2026. https://www.kcl.ac.uk/study/undergraduate/courses/dentistry-bds.

[61] Queen Mary University of London Dentistry BDS 2026. https://www.qmul.ac.uk/undergraduate/coursefinder/courses/2026/dentistry/.

[62] Association for Dental Education in Europe (ADEE) LEADER Programme 2026. https://adee.org/leader/leader-programme.

[63] Hugger A., Hugger S., Kordaß B. (2011). Die zahnärztliche Ausbildung. Bundesgesundheitsblatt-Gesundheitsforschung-Gesundheitsschutz.

[64] Olapade-Olaopa O., Adaramoye O., Raji Y., Fasola A.O., Olopade F. (2016). Developing a competency-based medical education curriculum for the core basic medical sciences in an African Medical School. Adv. Med. Educ. Pract..

[65] Postma T.C., White J.G. (2017). Students’ perceptions of vertical and horizontal integration in a discipline-based dental school. Eur. J. Dent. Educ..

[66] Al-Madi E.M., AlShiddi M., Al-Saleh S., AbdelLatif H. (2018). Developing a Dental Curriculum for the 21(st) Century in a New Dental School in Saudi Arabia. J. Dent. Educ..

