# Supplementary material for: Integrating Basic Sciences into Dental Education: A Comparative Narrative Review of Curricular Approaches Across Different Countries
Source: Dent J (Basel). 2026 Aug 19;14(8):529. doi: 10.3390/dj14080529 (PMC13510915; doi:10.3390/dj14080529)
Supplement: Supplementary file 1 [file dentistry-14-00529-s001.zip › dentistry-4411058-supplementary.pdf]

Table S1. Comparative summary of dental curricula across some of the included institutions. "-" indicates that the information was not reported in the available source(s). Source type is indicated to distinguish peer-reviewed evidence from institutional or personal-communication sources.

Country / Institution	Program duration	Timing of basic science teaching	Onset and form of clinical exposure	Dominant integration model	Pedagogical methods / key components	Challenging factors	Source type
**Sweden – Malmö University**	5-year undergraduate program	-	-	-	Problem-based learning<br>Challenge-based learning	Problem-based learning is demanding for the faculty, it requires strong institutional coordination and carries a risk of student overload	Peer-reviewed articles + Tandlægebladet
**Sweden – Karolinska Institutet**	5-year undergraduate program	-	First direct patient contact in 2nd year	Spiral integration	Workplace-based education in 9th-10th semester<br>Students from 10th semester act as supervisors for younger students	-	Personal communication
**Finland – University of Helsinki**	5.5-year undergraduate program	First 2 years	Early: 1-day orthodontist observation and assisting senior students in 1st year	Vertical integration	Simulation (from 1st–2nd year)<br>Problem-based learning<br>Dental-specific introductory courses in the early years, such as dental handcraft training	Student stress at the preclinical–clinical transition<br><br>Problem-based learning shared with medical students is valued less by dental students because dental perspectives are underrepresented	Personal communication + peer-reviewed articles
**Norway – University of Oslo**	5-year undergraduate program	First 2 years	First patient contact late in 5th semester (radiology only)<br>Regular clinical work from 6th semester	Horizontal integration	-	High student stress<br>Late clinical exposure	University webpage + peer-reviewed articles
**Norway – University of Bergen**	5-year undergraduate program	First 2 years	Assisting senior students in 2nd semester<br>First clinic patients in 5th semester	-	Patient-centered care	High student stress	University webpage + peer-reviewed articles
**Norway – The Arctic University of Norway**	5-year undergraduate program	First 2 years	Early and extensive: decentralized clinical rotations in rural areas	-	Decentralized rural rotations	High student stress	Peer-reviewed articles
**Denmark – Aarhus University**	5 years total:<br>3-year Bachelor of Science in Dental Surgery<br>2-year Master of Science in Dental Surgery	-	First patient contact in 3rd semester	-	Interdisciplinary work with technicians and hygienists<br>Early clinical exposure and simulation training<br>Emerging technologies in the program, such as the use of AI in radiology courses	-	Personal communication + University webpage + Tandlægebladet
**United States – University of Maryland**	4-year graduate-entry (Students must complete a bachelor's degree prior to admission)	First 2 years	First patient contact in 1st year	Spiral integration	Patient-centered and student-centered learning<br>Digital learning platforms and online activities	High need for faculty coordination	University webpage + peer-reviewed articles
**United States – Harvard University**	4-year graduate-entry (Students must complete a bachelor's degree prior to admission)	First 2 years	Emergent Clinical Experience in 2nd year (hospital/in-house rotations); own patients thereafter	Vertical integration	Case-based learning<br>Flipped classrooms<br>Early clinical exposure<br>Research project from 1st year	-	University webpage + peer-reviewed articles
**Canada – University of Toronto**	4-year graduate-entry (Students must complete a bachelor's degree prior to admission)	First 2 years	Assisting senior students in 1st–2nd year and extramural hospital rotations	Vertical and horizontal integration	Case-based learning	Financial/staffing sustainability	University webpage
**Canada – University of British Columbia**	4-year graduate-entry (Students must complete a bachelor's degree prior to admission)	-	2nd year in clinical groups	-	Hybrid case-based learning and problem-based learning<br>Clinical groups of students from different semesters	Students report high workload	Peer-reviewed articles + University webpage
**United Kingdom – King's College London**	5-year Bachelor of Dental Surgery program	First 2 years	Extensive patient contact from the 3rd year	Spiral integration	Patient-centered and evidence-based dentistry	-	University webpage + peer-reviewed articles

Country / Institution	Program duration	Timing of basic science teaching	Onset and form of clinical exposure	Dominant integration model	Pedagogical methods / key components	Challenging factors	Source type
**United Kingdom – Queen Mary University of London**	5-year Bachelor of Dental Surgery program	First 2 years	Early patient observation and clinical skills training in 1st year	Spiral integration	Interprofessional learning<br>Student-selected modules in areas of interest such as research, quality improvement projects and specialized clinical or theoretical subjects	-	University webpage
**Spain – Universitat Internacional de Catalunya**	5-year undergraduate program	1st year and part of 2nd year	Observation of final-year students in 3rd semester<br>And clinic in 4th semester	Vertical integration	Simulation (physical and digital)<br>No fixed preclinical training timeline, progression depends on the student's individual development	-	Personal communication
**Spain – Complutense University of Madrid**	5-year undergraduate program	First 2 years	Intensive clinical exposure in the 4th year	Largely sequential<br>Limited early vertical integration	-	Limited interdisciplinary approaches and early integration<br>Late clinical transition linked to lower confidence and higher stress	Personal communication
**China**	5+3 model:<br>5-year dental degree followed by a 3-year residency	First 2 years	Largely compressed into final-year clinical rotations	Traditional, discipline-based<br>Limited early integration	Exploration of problem-based learning and case-based learning	Clinical training is compressed, giving a heavy workload on students in the final year	Peer-reviewed articles
**Nigeria – University of Ibadan**	-	First 2 semesters	Early introduction via "Clinical Application of Basic Sciences" (co-taught) from 3rd semester<br>First patient contact is not reported	Vertical and horizontal integration	Competency-based education<br>Interprofessional learning<br>Problem-based learning	Implementation of the new curriculum faced resistance from both faculty members and students<br>Financial and infrastructure problems	Peer-reviewed articles
**Saudi-Arabia – Princess Nourah bint Abdulrahman University**	7 years total:<br>1-year foundation program<br>5-year dental education<br>1-year internship	First 2 years of the dental education	Early years: observation and preventive care<br>Last years: more patient care	Block- and stream courses-based<br>Vertical integration in later years	Problem- based learning<br>Interprofessional learning<br>Block- structured curriculum	High student stress<br>Course overlap<br>Faculty members need more student-centered pedagogical training	Peer-reviewed articles
